# Early prostate specific antigen decline and its velocity are independent predictive factors for outcomes of mCRPC patients treated with abiraterone acetate

**DOI:** 10.1186/s40779-021-00364-x

**Published:** 2022-01-24

**Authors:** Jiang-Yi Wang, Guo-Peng Yu, Long Li, Guo-Wen Lin, Ding-Wei Ye

**Affiliations:** 1 Department of Urology, Fudan University Shanghai Cancer Center, 270 Dong’an Road, Shanghai, 200032 China; 2 Department of Oncology, Shanghai Medical College, Fudan University, Shanghai, 200032 China; 3 Department of Urology, Shanghai Ninth People’s Hospital, Shanghai Jiaotong University School of Medicine, Shanghai, 200011 China

**Keywords:** Metastatic castration-resistant prostate cancer, PSA decline, PSA velocity, Overall survival, Time to PSA progression

Dear Editor,

Although the incidence of prostate cancer (PCa) has decreased in recent decades in Western countries, it has gradually increased in China due to the increasingly longer life expectancy and more popular westernized diet [1]. Androgen deprivation therapy (ADT) has been a cornerstone in the treatment of advanced PCa. However, most patients develop resistance to ADT within a median duration of 18–24 months, which is defined as castration-resistant prostate cancer (CRPC). Abiraterone acetate (AA), an inhibitor of the androgen biosynthesis enzyme CYP17 (17-a-hydroxylase and C17,20-lyase), has been approved for the treatment of metastatic CRPC, although not all patients benefit from AA treatment [2, 3]. Therefore, identifying patients who will respond to AA treatment and the time to second-line therapy is of great importance.

In this study, 120 Chinese patients diagnosed with metastatic castration-resistant prostate cancer (mCRPC) who were treated with AA at Fudan University Shanghai Cancer Center between April 2012 and February 2018 were recruited. Demographic characteristics, laboratory findings, and clinical outcomes were collected. Early prostate specific antigen (PSA) decline was examined at 4 weeks. The decreased PSA velocity (PSAV) during the first three months of treatment was calculated as the slope of the simple linear regression of PSA (natural scale) versus time. The Kaplan–Meier method with a log-rank test was performed to assess the overall survival (OS) and time to PSA progression (TTPP) of the two groups. Multivariate Cox regression models were used to identify independent risk factors for OS and TTPP. *P* < 0.05 (two sides) was considered statistically significant.

The baseline information is shown in Additional file 1: Table S1. The median PSA at diagnosis was 87.0 ng/ml. Patients in the pre-chemotherapy cohort showed a lower PSA value at diagnosis than those in the post-chemotherapy cohort (80.0 ng/ml vs. 120.5 ng/ml, *P* = 0.036, Additional file 1: Table S1). A total of 50 (41.7%) patients reached more than 30% PSA decline and 30 (25.0%) patients reached more than 50% decline at 4 weeks; the median PSAV during the first 12 weeks was − 0.77 ng/(ml∙month) (-8.33 to 8.63) (Additional file 1: Table S2).

A rapid PSA decrease during the first 12 weeks [PSAV > 0.77 ng/(ml∙month)] was associated with a longer OS (30.7 months vs. 22.1 months, *P* = 0.0023) (Additional file 2: Fig. S1 a-c) and a longer TTPP (22.7 months vs. 2.0 months, *P* < 0.001) than PSAV ≤ 0.77 ng/(ml∙month) (Additional file 2: Fig. S1 d-f). The multivariate Cox regression analysis showed that PSAV was an independent risk factor for OS and TTPP in mCRPC patients treated with AA (Additional file 1: Table S3). Meanwhile, patients with more than 30% early PSA decline showed a longer OS (29.7 months vs. 22.1 months, *P* = 0.02, Additional file 2: Fig. S1 g-i) and longer TTPP (22.4 months vs. 3.0 months, *P* < 0.001, Additional file 2: Fig. S1 j-l). In the multivariable Cox analysis, less than 30% early PSA decline and higher baseline PSA at the initiation of treatment were independent risk factors for OS and TTPP (Additional file 1: Table S4).

According to the Prostate Cancer Working Group 3 (PCWG3) criteria, 30% and 50% PSA declines at 12 weeks are commonly reported in clinical trials, and both are associated with improved survival [4]. In this study, we found that patients with 30% early PSA decline showed a longer OS and longer TTPP, which was in line with the findings of a UK cohort [5], and the results were stable for both pre-chemotherapy and post-chemotherapy cohorts. In addition, the PSA decline at 4 weeks was confirmed to be significantly correlated with the PSA change at 12 weeks (*r* = 0.82, *P* < 0.001) [5]. Considering that the clinical practice requires a biomarker of response as early as possible, we suggest that a 30% PSA decline at 4 weeks should be used as a reliable biomarker to predict the outcome of CRPC patients treated with AA before and after chemotherapy.

The other two predictors of OS were the lowest PSA during previous therapy and baseline PSA at AA initiation. The results suggest that the PSA nadir before AA and baseline PSA at AA initiation should be combined with an early PSA response to help physicians make earlier treatment change decisions. Future studies with a larger cohort are needed to evaluate the synergistic effect of PSA-related biomarkers before, during, and after AA treatment.

The limitations of this study include the retrospective nature and lack of external validation. Moreover, some new drugs that are not available in China have been approved in Western countries, leading to differences in therapeutic strategies between China and other countries. Thus, the conclusion of this study may not be applicable for all patients worldwide, but it will provide valuable information for the treatment of Chinese patients affected by PCa.

## Supplementary information

**Additional file 1: Table S1**. Characteristics of the mCRPC patients treated with abiraterone acetate (AA). **Table S2.** PSA changes among patients treated with abiraterone acetate (AA). **Table S3.** Multivariate COX regression analysis of PSAV for OS and TTPP. **Table S4.** Multivariate COX regression analysis of early PSA decline for OS and TTPP.

**Additional file 2: Fig. S1**. The predictive value of PSAV and early PSA change at 4 weeks for OS and TTPP of patients receiving abiraterone therapy. **a** PSAV for OS in overall cohort; **b** PSAV for OS in pre-chemotherapy cohort; **c** PSAV for OS in post-chemotherapy cohort. **d** PSAV for TTPP in overall cohort; **e** PSAV for TTPP in pre-chemotherapy cohort; **f** PSAV for TTPP in post-chemotherapy cohort. **g** Early PSA decline for OS in overall cohort; **h** Early PSA decline for OS in pre-chemotherapy cohort; **i** Early PSA decline for OS in post-chemotherapy cohort. **j** Early PSA decline for TTPP in overall cohort; **k** Early PSA decline for TTPP in pre-chemotherapy cohort; **l** Early PSA decline for TTPP in post-chemotherapy cohort. OS overall survival, PSA prostate specific antigen, PSAV prostate specific antigen velocity, TTPP time to PSA progression

## Data Availability

The data supporting the conclusions of this article are included within the article.

## Abbreviations

AA: Abiraterone acetate
ADT: Androgen deprivation therapy
ALP: Alkaline phosphatase
CRPC: Castration-resistant prostate cancer
LDH: Lactate dehydrogenase
mCRPC: Metastatic castration-resistant prostate cancer
OS: Overall survival
PCa: Prostate cancer
PCWG: Prostate Cancer Working Group
PSA: Prostate-specific antigen
PSAV: PSA velocity
TTPP: Time to PSA progression
