# Supplementary material for: Early prostate specific antigen decline and its velocity are independent predictive factors for outcomes of mCRPC patients treated with abiraterone acetate
Source: Mil Med Res. 2022 Jan 24;9:5. doi: 10.1186/s40779-021-00364-x (PMC8785553; doi:10.1186/s40779-021-00364-x)
Supplement: Supplementary file 1 — Additional file 1: Table S1. Characteristics of the mCRPC patients treated with abiraterone acetate (AA). Table S2. PSA changes among patients treated with abiraterone acetate (AA). Table S3. Multivariate COX regression analysis of PSAV for OS and TTPP. Table S4. Multivariate COX regression analysis of early PSA decline for OS and TTPP. [file 40779_2021_364_MOESM1_ESM.pdf]

Table S1 Characteristics of the mCRPC patients treated with abiraterone acetate (AA)

Item	Overall (n = 120)	Pre-chemotherapy (n = 88)	Post-chemotherapy (n = 32)	P
PSA at diagnosis [ng/ml, M (Q_1, Q_3)]	87.0(33.8-179.4)	80.0(30.6-151.5)	120.5(56.3-350.8)	0.036
Gleason score [n(%)]				
<8	28(23.3)	23(26.1)	5(15.6)	0.229
≥8	92(76.7)	65(73.9)	27(84.4)	
Lowest PSA [ng/ml, M (Q_1, Q_3)]	0.60(0.06-4.00)	0.38(0.05-3.12)	1.23(0.10-9.83)	0.085
LN metastasis [n(%)]				
Yes	27(22.5)	19(21.6)	8(22.5)	0.692
No	93(77.5)	69(78.4)	24(77.5)	
Painkiller [n(%)]				
Yes	15(12.5)	13(14.8)	2(6.3)	0.359
No	105(87.5)	75(85.2)	30(93.8)	
Baseline PSA at AA use [ng/ml, M (Q_1, Q_3)]	41.30 (10.89-155.00)	36.37 (9.87-139.58)	70.95 (12.10-253.45)	0.144
Testosterone [ng/ml, M (Q_1, Q_3)]	0.13(0.13-0.32)	0.14(0.13-0.32)	0.13(0.13-0.29)	0.995
ALP [U/L, M (Q_1, Q_3)]	101.80 (68.10-190.50)	113.70 (70.30-200.00)	88.10 (57.48-165.88)	0.441
LDH [U/L, M (Q_1, Q_3)]	219.70 (177.30-324.50)	227.60 (189.30-330.30)	199.50 (159.30-294.50)	0.125

ALP alkaline phosphatase; *LDH* lactate dehydrogenase; *LN* Lymph node; *mCRPC* metastatic castration-resistant prostate cancer; *PSA* prostate specific antigen

Table S2 PSA changes among patients treated with abiraterone acetate (AA)

Outcome	Overall (n = 120)	Pre-chemotherapy (n = 88)	Post-chemotherapy (n = 32)	P
30% decline at 4 weeks [n (%)]				0.577
Yes	50(41.7)	38(43.2)	12(37.5)	
No	70(58.3)	50(56.8)	20(62.5)	
50% decline at 4 weeks [n (%)]				0.057
Yes	30(25.0)	26(29.5)	4(12.5)	
No	90(75.0)	62(70.5)	28(87.5)	
PSA progression at 4 weeks [n (%)]				0.006
Yes	37(30.8)	21(23.9)	16(50.0)	
No	83(69.2)	67(76.1)	16(50.0)	
PSAV during first 12 weeks [ng/(ml·month), M (Q_1, Q_3)]	-0.77(-8.33 to 8.63)	-1.25(-8.33 to 2.45)	6.31(-4.71 to 78.68)	0.032

PSA prostate specific antigen; *PSAV* prostate specific antigen velocity

Table S3 Multivariate COX regression analysis of PSAV for OS and TTPP

Variables	OS			TTPP		
	HR	95% CI	*P*	*HR*	95% CI	*P*
PSAV [≤0.77 ng/(ml·month) *vs*. > 0.77 ng/(ml·month)]	2.468	1.373-4.434	0.003	6.492	3.809-11.064	<0.001
PSA at diagnosis	1.000	0.994-1.000	0.304	1.000	1.000-1.000	0.363
Gleason score	1.126	0.844-1.503	0.418	1.022	0.818-1.277	0.848
Chemotherapy (Yes *vs*. No)	1.400	0.747-2.617	0.294	2.186	1.283-3.726	0.004
Lowest PSA	1.008	1.000-1.017	0.045	1.002	0.994-1.010	0.702
Lymph node metastasis (Yes *vs*. No)	0.933	0.468-1.858	0.843	1.188	0.662-2.135	0.564
Painkiller (Yes *vs*. No)	2.981	1.447-6.142	0.003	1.392	0.730-2.656	0.315
Baseline PSA at AA use	1.001	1.001-1.002	<0.001	1.001	1.000-1.001	0.023
Testosterone	0.877	0.742-1.039	0.130	0.911	0.795-1.044	0.179
ALP	1.001	1.00-1.002	0.042	1.000	0.999-1.001	0.680
LDH	1.000	0.998-1.001	0.497	1.000	0.998-1.001	0.423

AA abiraterone acetate; *ALP* alkaline phosphatase; *LDH* lactate dehydrogenase; *OS* overall survival; *PSA* prostate specific antigen; *PSAV* prostate specific antigen velocity; *TTPP* time to PSA progression

Table S4 Multivariate COX regression analysis of early PSA decline for OS and TTPP

Variables	OS			TTPP		
	HR	95% CI	*P*	*HR*	95% CI	*P*
PSA decline (< 30% *vs*. ≥ 30%)	2.174	1.148-4.120	0.017	3.526	2.078-5.983	< 0.001
PSA at diagnosis	1.000	0.999-1.000	0.317	1.000	1.000-1.000	0.463
Gleason score	1.127	0.848-1.498	0.409	1.047	0.836-1.310	0.690
Chemotherapy (Yes *vs*. No)	1.450	0.785-2.679	0.235	1.679	1.021-2.761	0.041
Lowest PSA	1.012	1.004-1.021	0.004	1.007	0.999-1.016	0.100
Lymph node metastasis (Yes *vs*. No)	0.779	0.390-1.559	0.481	0.829	0.460-1.496	0.534
Painkiller (Yes *vs*. No)	2.986	1.481-6.020	0.002	1.600	0.844-3.034	0.150
Baseline PSA at AA use	1.001	1.001-1.002	< 0.001	1.001	1.000-1.001	0.040
Testosterone	0.898	0.765-1.054	0.187	0.917	0.807-1.043	0.187
ALP	1.001	1.00-1.002	0.066	1.000	0.999-1.001	0.835
LDH	1.000	0.999-1.002	0.947	1.000	0.999-1.002	0.357

AA abiraterone acetate; *ALP* alkaline phosphatase; *LDH* lactate dehydrogenase; *OS* overall survival; *PSA* prostate specific antigen; *TTPP* time to PSA progression
