# Supplementary material for: Early prostate specific antigen decline and its velocity are independent predictive factors for outcomes of mCRPC patients treated with abiraterone acetate
Source: Mil Med Res. 2022 Jan 24;9:5. doi: 10.1186/s40779-021-00364-x (PMC8785553; doi:10.1186/s40779-021-00364-x)
Supplement: Supplementary file 2 — Additional file 2: Fig. S1. The predictive value of PSAV and early PSA change at 4 weeks for OS and TTPP of patients receiving abiraterone therapy. a PSAV for OS in overall cohort; b PSAV for OS in pre-chemotherapy cohort; c PSAV for OS in post-chemotherapy cohort. d PSAV for TTPP in overall cohort; e PSAV for TTPP in pre-chemotherapy cohort; f PSAV for TTPP in post-chemotherapy cohort. g Early PSA decline for OS in overall cohort; h Early PSA decline for OS in pre-chemotherapy cohort; i Early PSA decline for OS in post-chemotherapy cohort. j Early PSA decline for TTPP in overall cohort; k Early PSA decline for TTPP in pre-chemotherapy cohort; l Early PSA decline for TTPP in post-chemotherapy cohort. OS overall survival, PSA prostate specific antigen, PSAV prostate specific antigen velocity, TTPP time to PSA progression [file 40779_2021_364_MOESM2_ESM.pdf]

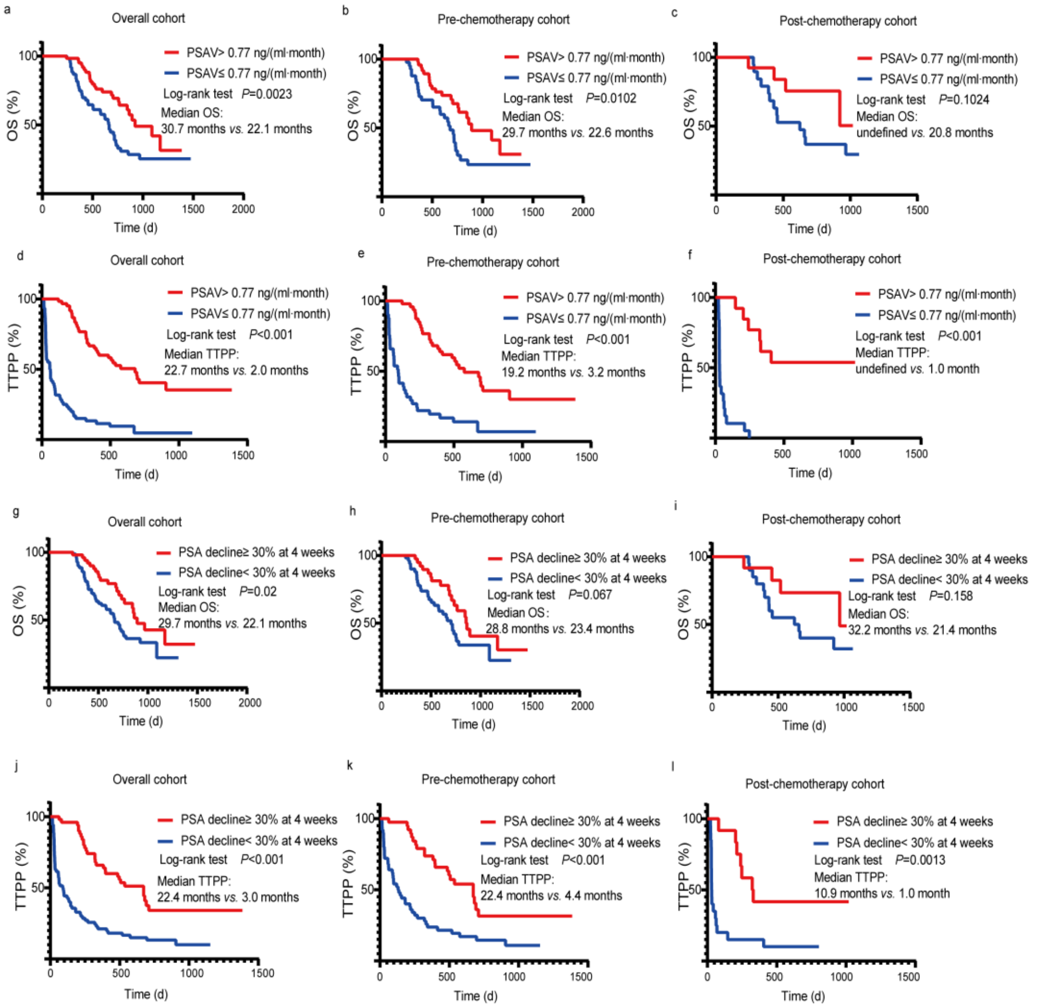

Fig. S1 The predictive value of PSAV and early PSA change at 4 weeks for OS and TTPP of patients receiving abiraterone therapy. **a** PSAV for OS in overall cohort; **b** PSAV for OS in pre-chemotherapy cohort; **c** PSAV for OS in post-chemotherapy cohort. **d** PSAV for TTPP in overall cohort; **e** PSAV for TTPP in pre-chemotherapy cohort; **f** PSAV for TTPP in post-chemotherapy cohort. **g** Early PSA decline for OS in overall cohort; **h** Early PSA decline for OS in pre-chemotherapy cohort; **i** Early PSA decline for OS in post-chemotherapy cohort. **j** Early PSA decline for TTPP in overall cohort; **k** Early PSA decline for TTPP in pre-chemotherapy cohort; **l** Early PSA decline for TTPP in post-chemotherapy cohort. OS overall survival, PSA prostate specific antigen, PSAV prostate specific antigen velocity, TTPP time to PSA progression
